# Usefulness and Clinical Impact of Whole-Body MRI in Detecting Autoimmune Neuromuscular Disorders

**DOI:** 10.3390/brainsci13101500

**Published:** 2023-10-23

**Authors:** Mario Pace, Roberto Cannella, Vincenzo Di Stefano, Antonino Lupica, Paolo Alonge, Giulio Morici, Filippo Brighina, Federica Brancato, Federico Midiri, Massimo Galia

**Affiliations:** 1 Section of Radiology, Department of Biomedicine, Neuroscience and Advanced Diagnostics (BiND), University Hospital “Paolo Giaccone”, Via del Vespro 129, 90127 Palermo, Italy; mario.pace@community.unipa.it (M.P.); roberto.cannella@unipa.it (R.C.); giulio.morici@community.unipa.it (G.M.); 2 Section of Neurology, Department of Biomedicine, Neuroscience and Advanced Diagnostic (BIND), University of Palermo, 90127 Palermo, Italy; vincenzo.distefano07@unipa.it (V.D.S.); antonino.lupica@unipa.it (A.L.); alongep95@gmail.com (P.A.); filippo.brighina@unipa.it (F.B.); 3 Department of Surgical, Oncological and Oral Science (Di.Chir.On.S.), University of Palermo, 90133 Palermo, Italy; federica.brancato91@gmail.com; 4 IRCCS Ospedale Galeazzi-Sant’Ambrogio, Via Cristina Belgioioso 173, 20161 Milano, Italy; federico.midiri@hotmail.com

**Keywords:** whole-body MRI, autoimmune neuromuscular disorder, AIDP, CIDP, myasthenia gravis, Lambert–Eaton, idiopathic inflammatory myopathies, focal myositis

## Abstract

Autoimmune neuromuscular diseases are a group of heterogenous pathologies secondary to the activation of the immune system that damage the structures of the peripheric nerve, the neuromuscular junction, or the skeleton muscle. The diagnosis of autoimmune neuromuscular disorders comprises a combination of data from clinical, laboratory, electromyography, imaging exam, and biopsy. Particularly, the whole-body MRI examination in the last two decades has been of great use in the assessment of neuromuscular disorders. MRI provides information about the structures involved and the status of activity of the disease. It can also be used as a biomarker, detect the pattern of specific muscle involvement, and is a useful tool for targeting the optimal muscle site for biopsy. In this work, we summarized the most used technical protocol of whole-body MRI and the role of this imaging technique in autoimmune neuromuscular disorders.

## 1. Introduction

In the broad spectrum of neuromuscular disorders, autoimmune neuromuscular diseases are a group of heterogenous pathologies secondary to immune system activation, damaging the structures of the peripheric nerve (i.e., acute inflammatory demyelinating polyneuropathy (AIDP) or Guillen–Barré syndrome, and chronic inflammatory demyelinating polyneuropathy (CIDP)) [1], neuromuscular junction (i.e., myasthenia gravis, Lambert–Eaton myasthenic syndrome (LEMS)) [2] or the skeleton muscle (i.e., idiopathic inflammatory myopathies (IIM)) [3].

The diagnosis of autoimmune neuromuscular disorders comprises a combination of clinical symptom onset and progression, an evaluation of serum antibodies and markers (creatine kinase, CK), electromyography, and biopsy [2,4].

In the last two decades, musculoskeletal imaging has become relevant in the diagnostic work-up of neuromuscular diseases. Among the different imaging modalities, magnetic resonance imaging (MRI) has the advantage of better contrast and a lack of ionizing radiation [5]. In addition, MRI can provide information about the status of activity of the disease in the muscle [6] and nerve [7], detecting patterns of specific muscle involvement that might point to a diagnosis [8]. MRI has also the advantage of targeting the optimal muscle for biopsy, especially for the IIM [9], and it represents a useful biomarker for response to the treatment [10].

Particularly, the whole-body MRI examination has been recently employed for neuromuscular disorders [11]. 

Whole-body MRI allows for the multiparametric imaging assessment of all muscular structures in a single examination with narrow scanning times [12]. 

Moreover, whole-body MRI has a high sensitivity, revealing the global distribution of muscle involvement and detecting the involvement of certain muscles clinically silent [13].

In this review, we summarized the most used technical protocol of whole-body MRI and the role of this imaging technique in the diagnosis, prognosis, monitoring of disease progression, and quantification of response to treatment in patients with neuromuscular diseases [14].

## 2. MRI Whole-Body Protocols 

Whole-body MRI for the neuromuscular disorder is technically feasible on both 1.5 T and 3 T MR scanners. The use of 3 T MR improves the signal-to-noise ratio (SNR) and image quality. The MR scanners should be equipped with a moving table, allowing for the optimal placement of the patient body in the magnet bore. 

The patient lays in a supine position, with arms resting by their sides and hands, aligned in the sagittal plane [15]. 

To obtain high-spatial-resolution images, sequential acquisitions in limited parts of the body (head–neck–trunk, abdomen, thighs, and legs–feet) are needed, with the magnet in each scansion isocenter employed by successive or continuous table motions [16]. 

To better quantify the muscle edema and the fatty involvement, a minimum slice thickness of 5 to 8 mm, with an interslice gap of 1 to 2 mm maximum, is recommended [5]. Surface coils are placed, covering the head and neck, chest, abdomen, and lower limbs, to improve the SNR [17]. 

The acquisition should include the whole body, from head to feet, with a sequential acquisition of the head, trunk, abdomen, thighs, legs, and feet. During thoracic and abdominal acquisition, coordinated breathing and controlled apneas (if tolerated by the patient) allow for a reduction in artifacts from respiratory movements [18]. 

It is possible to omit the chest with a markedly reduced overall acquisition time and a similar diagnostic accuracy for inflammatory myopathy [19]. 

Notwithstanding the numerous advantages of muscle MRI, this technique is not widely available, being performed only in selected centers, and a standardized MRI protocol for autoimmune neuromuscular disorders is still lacking.

**Figure 1:**
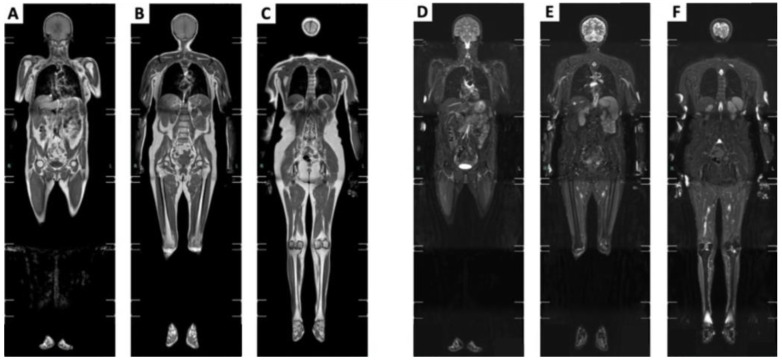
Coronal composite views generated by combining the series of coronal T1-weighted and STIR images. (**A**–**C**) Coronal compositive view, T1-weighted, sliding anterior to posterior side, respectively. (**D**–**F**) Coronal compositive view, STIR, sliding anterior to posterior side, respectively.

Suggested protocols for whole-body MRI should include axial T1-weighted and STIR images to asset qualitative muscle volume and fatty tissue replacement in muscle or edema, respectively [20]. Coronal STIR and T1-weighted images should be acquired, and the successive series of images should be automatically combined to generate a single coronal composite view [11] (Figure 1).

To obtain a semiquantitative asset of the fat content of the muscle, it is possible to use the Dixon method with the fat fraction value [21]. 

A functional MRI technique such as Diffusion Weighted Imaging (DWI) is required in the protocol, characterizing the cellularity, the water content, and fibrosis of the tissue. DWI analyzes the stochastic Brownian motion of extracellular water molecules within tissues using a different gradient; for the study of the neuromuscular disorder gradient, a b-value of 400–800 is sufficient. 

In a separate study, DWI increased in active inflammation and decreased in the case of fatty degeneration [22]. In addition, DWI can be useful in detecting some areas suspected of heteroplasia. 

The acquisition time for all examinations is around 50 min. 

**Figure 2:**
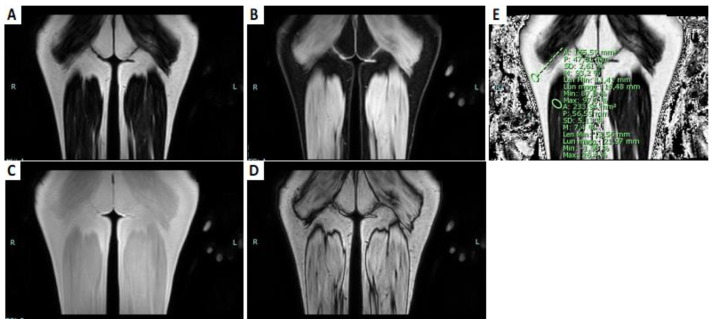
T1-weighted DIXON sequences of the posterior part of the thighs. (**A**) Fat-only coronal image. (**B**) Water-only coronal image. (**C**) Coronal T1-weighted in phase image. (**D**) Coronal T1-weighted out-phase image. (**E**) Coronal fat fraction map; the sites of ROIs: one in the muscular belly of the long head of the right biceps femoris; the other in the subcutaneous fat.

Using the new Dixon sequences, it is possible to reduce the time of acquisition by acquiring more echoes (such as three-point Dixon or six-point Dixon); it is also possible to improve the SNR, possessing in only one acquisition the specific fluid-sensitive images (water-only), fat-sensitive images (fat-only), and T2* (T2 Star) images. Furthermore, it is possible to make reconstructions in all spatial planes due to the isometric voxel, and provide water and fat separation with a correction of the T2* effect—producing the proton density fat fraction (FF)—without error, due to the field inhomogeneity of iron [23] (Figure 2).

In inflammatory neuropathies, additional neurographies sequences [24] or the diffusion tensor imaging of peripheral nerves are required to quantify nerve damage and regeneration [7]. Postcontrast sequences and diffusion-weighted imaging are not mandatory, because these sequences have limited use in the diagnosis of inherited myopathies [16]. Indeed, the use of intravenous contrast medium gadolinium-enhanced fat-suppressed T1-weighted imaging (Gd-T1w) is still controversial. Gadolinium uptake was described in the inflammation area of the muscle, typical of idiopathic inflammatory myopathies (i.e., dermatomyositis) [25,26], but was observed less frequently than edema, even in the case of myopathies. These findings underline the limited value of gadolinium in suspected myopathies [27].

## 3. Whole-Body MRI: Qualitative Assessment 

The possibility to have a whole-body acquisition allows for an immediate comparison of muscles affected and spared by the disease process, the pattern (i.e., proximal, distal, facial, pharyngeal, etc.), the symmetry (i.e., right- or left-predominant), the muscle mass representation (i.e., grade of hypotrophy, pseudohypertrophy, etc.), and the presence of inflammation, fibrosis, or adipose substitution.

### 3.1. Distinction between Active and Chronic Disease

#### 3.1.1. Acute Changes

Acute changes are characterized by edema-like changes in the muscle, evaluated on STIR or T2-weighted images with a suppressed fat signal. It appears as focal or a diffuse hyperintensity of the involved skeletal muscles [28]. Intramuscular edema is non-specific and may also be found after trauma or overuse of the muscle in infective and inflammatory myopathies, neoplasm, rhabdomyolysis, recent vascular events, and even denervation (focal neuropathy) [29,30,31].

Muscle edema can be classified based on hyperintensity on STIR sequences with a semi-quantitative three-point scale [32]:

0 = Normal muscle intensity;
1 = Mild hyperintensity;
2 = Definite hyperintensity.

Is important to report if the edema is confined only in the muscle or is also present in the cutaneous and subcutaneous tissue and in the pattern of muscle edema because it may help in narrowing the diagnostic hypothesis toward certain pathologies, especially in idiopathic inflammatory myopathies.

**Figure 3:**
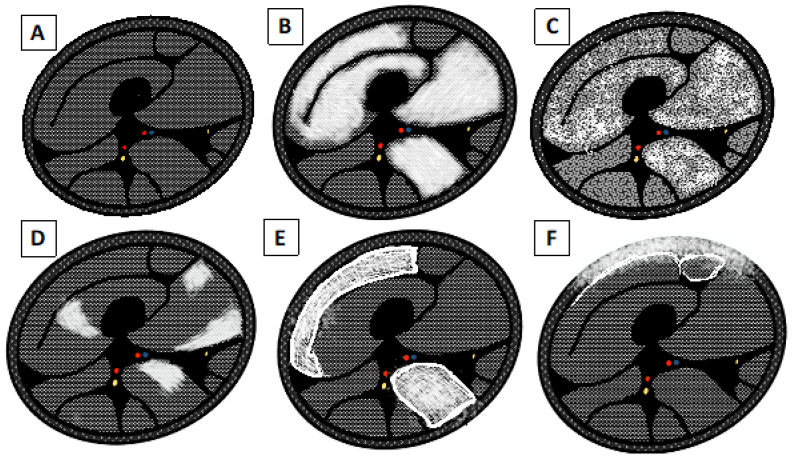
Schematic drawing of pattern edema. (**A**) Normal appearance of the muscle belly. (**B**) Diffuse end homogeneous edema. (**C**) “Foggy” edema. (**D**) Focal, “patchy” edema. (**E**) Subfascial and interfascicular edema with reticular or “honeycombing” appearance. (**F**) Subcutaneous and subfascial edema.

Regarding the types of muscle edema pattern (Figure 3), it is possible to see diffuse edema when: the entire muscle is affected by the edema, showing a very high intensity; “foggy” edema, where there is homogeneous moderate hyperintensity that does not hide the vessels’ backgrounds; patchy edema, affecting some point of the muscle; and reticular or honeycombing edema [26].

In addition, fluid-sensitive sequences provide further information through the form and anatomic distribution of the pathology, enabling the identification of the muscles most suitable for biopsy [9,33].

#### 3.1.2. Chronic Changes

Chronic disease results in a change of muscle size or shape and loss of contractile units usually replaced by fat that can be evaluated on the T1-weighted images without fat saturation [34]. 

**Figure 4:**
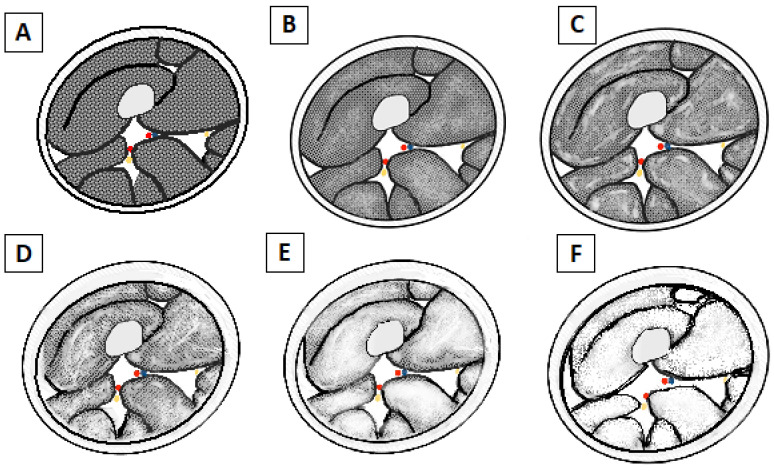
Schematic drawing of Mercuri’s classification of fatty atrophy grade. (**A**) Grade 0: Normal appearance of the muscle belly. (**B**) Grade 1: Early moth-eaten appearance with scattered small areas of fatty replacement. (**C**) Grade 2a: Late moth-eaten appearance, with numerous areas of fatty replacement with beginning confluence comprising less than 30%. (**D**) Grade 2b: Late moth-eaten appearance, with numerous areas of fatty replacement, with beginning confluence comprising 30–60%. (**E**) Grade 3: Washed-out, fuzzy appearance due to confluent areas of fatty replacement, with muscle still present at the periphery. (**F**) Grade 4: End-stage appearance, all muscle replaced by fat with complete fatty replacement.

**Figure 5:**
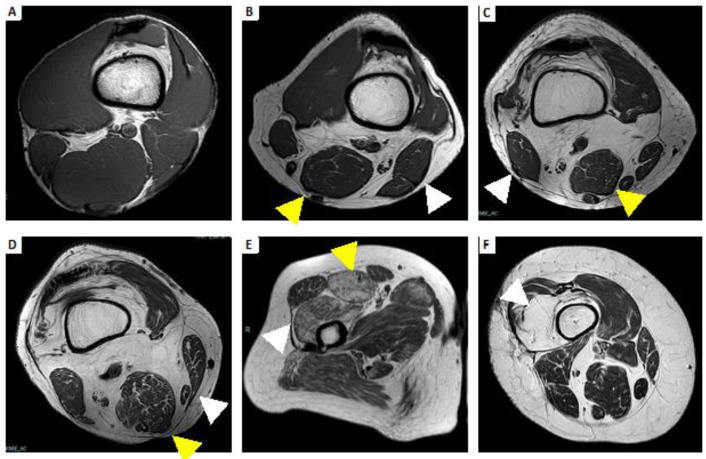
Example atrophy with fatty replacement using Mercuri’s classification. (**A**) Grade 0: All the muscles have regular intensity on T1-weighted imaging. (**B**) Grade 1: Fatty atrophy of semimembranosus (yellow arrowhead) and biceps femoris (white arrowhead), showing early moth-eaten appearance with scattered small areas of high signal on T1-weighted imaging. (**C**) Grade 2a: Fatty atrophy of semimembranosus (yellow arrowhead) and biceps femoris (white arrowhead), showing late moth-eaten appearance, with numerous areas of high signal on T1-weighted imaging, with beginning confluence comprising less than 30%. (**D**) Grade 2b: Fatty atrophy of semimembranosus (yellow arrowhead) and sartorius (white arrowhead), showing late moth-eaten appearance, with numerous areas of high signal on T1-weighted image, with beginning confluence comprising 30–60%. (**E**) Grade 3: Fatty atrophy of rectus femoris (yellow arrowhead) and vastus lateralis and vastus intermedius (white arrowhead), showing a fuzzy appearance due to confluent areas of high signal on T1-weighted imaging, with muscle still present at the periphery. (**F**) Grade 4: Fatty atrophy of vastus intermedius (white arrowhead); all muscle replaced by fat shows a homogeneous high signal on T1-weighted imaging.

T1-weighted images can provide semi-quantitative information about fat replacement, using the Mercuri classification (Figure 4 and Figure 5), which is an MRI-adaptation of the CT classification of Goutallier and Bernageau, allowing us to estimate the degeneration of the shoulder rotators [35], and it is divided into four stages [36]:

0 = Normal intensity appearance of the muscle.
1 = Early moth-eaten appearance with scattered small areas of high intensity on T1-weighted imaging.
2a = Late moth-eaten appearance, with numerous areas of high intensity on T1-weighted imaging, with beginning confluence comprising less than 30%.
2b = Late moth-eaten appearance, with numerous areas of high intensity on T1-weighted imaging, with beginning confluence comprising 30–60%.
3 = Washed-out, fuzzy appearance due to confluent areas of increased intensity in T1-weighted imaging, with muscle still present at the periphery.
4 = End-stage appearance; all muscle replaced by fat with high intensity in T1-weighted.

In atrophic striated skeletal muscle, the substitution of contractile fibers can be made not only with fat degeneration, but also by connective tissue, mainly collagen [37]. Fibrosis, more than fatty infiltration, has a deleterious effect on the loss of muscle function [38]. Endomysial fibrosis is a histopathologic parameter significantly correlated with poor motor outcome [39].

It is hard to present a direct qualitative assessment of the fibrous degeneration of the skeletal muscle. Because collagen has a very short T2 time, in the range of one second to a few hundred μs, the signal of these spins is phased out and does not contribute to the image [40]. Therefore, when collagen is grouped in connective tissue, such as tendons or scars, a low signal intensity appears in both weighted images. Conversely, when collagen is in the range of a few percent of muscle volume, it appears to be hidden by the spatial dependence of tissue signal to voxel position.

There are different experimental methods to quantify muscle fibrosis such as MR elastography, magnetization transfer, and sodium imaging, but more clinical validation remains necessary [41].

The indirect evaluation of the fibrous involution of the muscle can be determined by the loss of muscle volume [42].

### 3.2. Biomarker for the Evaluation of the Treatment and Follow-Up (Quantitative Assessment)

Whole-body MRI might become an excellent noninvasive and complete tool for evaluating the quantitative and objective fatty atrophy and activity of the muscle. 

In the past, proton spectroscopy, with the limitations of low spatial resolution and long acquisition time, was used for the fat/water fraction measurement; now, the Dixon technique is used, which has the same capabilities of spectroscopy, with high spatial resolution [43]. The Dixon technique provides water and fat distribution maps to suppress the fat signal or water signal, respectively, in post-processing and not during acquisition, such as other fat saturation techniques [44,45].

Quantitative biomarkers of chronic degenerative change are muscle fat fraction (MFF), muscle fat infiltration (MFI), and lean muscle. MFF is defined by the ratio between the volume of fat within the muscle and total muscle volume [46]. 

Using Dixon sequences, it is also possible to quantify the MFI by measuring the fat fraction contained in the muscle. 

Lean muscle volume is obtained by subtracting the fat volume from the entire muscle volume [47].

The images weighed in T2 can provide information on the presence of water and therefore edema in the muscle, quantifying the inflammation state of the same. But T2 time measurement can be influenced by the fat replacement signal of the muscle. Therefore, it is possible to avoid this error using a fat-corrected T2 map (fc-T2 Map). The fc-T2 map is obtained after fat fraction is calculated from the in- and out-of-phase gradient echo images based on a Dixon formulation [48].

## 4. Whole-Body MRI in Inflammatory Neuromuscular Disease

Characteristic patterns found in the whole-body MRI can help, together with clinical data, laboratory, and other diagnostic tests, to arrive at a diagnosis, or at least to narrow the diagnostic possibilities [49].

### 4.1. Guillan–Barré Syndrome

Among the subtypes of Guillan–Barré such as acute motor axonal neuropathy (AMAN) and acute motor and sensory axonal neuropathy (AMSAN), acute inflammatory demyelinating polyradiculoneuropathy (AIDP) is the most frequent [50,51]. 

AIDP is a peripheral neuropathy caused by demyelination due to the activation of T-lymphocytes that activate a cellular-mediated response with macrophages that degrade the myelin [52]. 

The extension and distribution of inflammation correspond to the clinical deficit [53]. Most cases are idiopathic, but some cases have been associated with post-infection due to a cross-reaction with axolemma or Schwann antigens [49]. Common symptoms are pain, numbness, and paresthesia that usually begin proximally and then proceed distally. The involvement of facial nerves is also described, and less often, the bulbar and ocular motor nerves. Autonomic involvement that manifest as urine retention, ileus, sinus tachycardia, hypertension, cardiac arrhythmia, and postural hypotension is common. 

AIDP has been arbitrarily defined when the onset phase lasts up to 4 weeks [54]. 

MRI, on the other hand, is not part of the routine diagnostic test. 

In acute GBS, muscles typically exhibit no changes in fluid-sensitive sequences unless there is an acute denervation damage [55]. In the study by Jinfeng Cao et al., the evaluation of the DTI of tibial and common peroneal nerves in patients with Guillain–Barré syndrome demonstrated that these sequences can provide important quantitative parameters for the evaluation of the peripheral nerve damage in patients with GBS, showing a moderate correlation between DTI and electrophysiology parameters [56].

A typical sensitive but non-specific feature is the presence of nerve root enhancement on gadolinium-enhanced MRI [57].

But the spatial resolution of the column in the whole-body MRI is lower than the sequences with FOV aimed at the study of the column. Moreover, the diagnosis of GBS is essentially clinical, electrophysiological, and laboratory. 

Therefore, MRI whole-body finds little indication in clinical practice.

### 4.2. Chronic Inflammatory Demyelinating Polyneuropathy

Chronic inflammatory demyelinating polyneuropathy (CIDP) is an acquired immune-mediated disease characterized by lymphocyte infiltration that damages the myelin of the nerves [58]. CIPD can cause a group of neuromuscular disorders that present chronic and progressive symptoms, over a span of more than 8 weeks. The disease pathogenesis remains unknown [59]. Clinically, it is characterized by a progressive or relapsing–remitting weakness of proximal and distal muscles with areflexia and paresthesia, but the diagnosis is difficult and there are many atypical variants [60,61].

The second revision of the Taskforce made by the European Federation of Neurological Societies and Peripheral Nerve Society (EFNS/PNS) and published in 2021 established that clinical and electromyographic tests are the most important diagnostic criteria that can differentiate between typical CIDP and CIDP variants. MRI and others diagnostic tests such as laboratory, cerebrospinal fluid analysis, ultrasound of the peripherical nerve, nerve biopsy, and treatment response are used as supportive diagnostic tools [62].

MRI might show a marked thickening of the spinal nerve roots and their peripheral nerves in the lumber and brachial plexuses, which are strongly supportive of the diagnosis of CIDP with a prolonged clinical course [1,63]. Whole-body MRI shows the edema or the fatty involution in the belly of the muscles innervated by the nerve affected. Therefore, whole-body MRI using sequences such as diffusor tensor imaging for the evaluation of the peripherical nerve and quantifying the fatty infiltration in the muscle with MFF is becoming a useful tool for the evaluation of response to the therapy of patients with CIDP [7].

Whole-body MRI has not yet entered the clinical practice for the management of this pathology. In the future, it will be assigned a research field and will certainly have a role, especially in characterizing the atypical forms of CIDP.

### 4.3. Myasthenia Gravis

Myasthenia gravis (MG) is the most common autoimmune disorder that affects the neuromuscular junction. It affects more women than men under 40 years of age, with a ratio of 3:1; after 50 years of age, men are more affected [64]. It can be correlated to thymus pathology [65], thyroid disorder [66], and other autoimmune diseases (i.e., systemic lupus erythematosus or rheumatoid arthritis) [67].

It is considered a classic example of an antibody-mediated disease because it is characterized by the presence of antibodies against the receptor of acetylcholine (AChR) on the post-synapse membrane of the muscle [68]. 

There are also other forms of myasthenia gravis with antibodies directed to other structures, such as anti-MuSK (muscle-specific kinase) antibodies [69], anti-Lrp4 (low-density lipoprotein receptor-related protein 4) antibodies [70], or anti-agrin antibodies [71]. 

MG is characterized by weakness that worsens with activity but improves with rest. Onset usually presents ptosis, diplopia, and ophthalmoplegia due to the involvement of the extra-orbital muscles in the eyes. Then, the disease progresses with dysarthria, fatigable mastication, and weakness in the limb and neck [72]. 

Myasthenia gravis crisis is a severe complication seen in 15–20% of patients with anti-AChR antibodies, manifesting with respiratory weakness and thus requiring mechanical ventilation support [73,74].

Imaging is useful in detecting thymus pathology. Chest CT is an efficient imaging tool to detect thymoma, while MRI shows high specificity and sensitivity to also detect thymus hyperplasia, and it is a useful tool for post-operative follow-up [65]. 

The limit of whole-body MRI in assessing myasthenia gravis is its poor spatial resolution for evaluating retroorbital muscles. In this case, the MRI focused on the orbital region is an excellent imaging tool for the evaluation of extra-orbital muscle involvement [75].

To the best of our knowledge, there is no article about the use of whole-body MRI in acquired myasthenia gravis. In one study, Finlayson et al. demonstrate the useful role of the whole-body MRI in investigating the congenital myasthenia gravis differencing pattern of muscle involvement [76]. 

The presence of anti-titin antibodies is frequently associated between myasthenia gravis and polymyositis [77]. In this case, whole-body MRI can be useful.

### 4.4. Idiopathic Inflammatory Myopathies 

Idiopathic inflammatory myopathies (IIMs) are a heterogeneous group of autoimmune diseases that primarily affect the skeletal muscles, but can also involve the skin, the lungs, the heart, and the gastrointestinal system [78]. 

The five most recognized types of inflammatory myopathies are dermatomyositis, polymyositis, immune-mediated necrotizing myopathy, overlap myositis (including antisynthetase syndrome), and sporadic inclusion body myositis [79,80]. 

IMMs can also occur, in the context of “overlap myositis”, in certain connective tissue diseases (CTDs) such as systemic sclerosis (SSc) and in patients with other rheumatic diseases such as rheumatoid arthritis, primary Sjogren’s syndrome, or systemic lupus erythematosus. Antinuclear antibodies (ANA) are more commonly detected in patients with IIMs [81].

A correlation between IMMs and interstitial lung disease (ILD) is known, especially in patients with the positivity of antisynthetase autoantibodies, such as anti-Jo-1, anti-PL-12 (anti-alanyl tRNA synthetase), and anti-PL-7 (anti-threonyl tRNA synthetase) that are strongly associated with ILD [82]. 

In this case, whole-body MRI can lead to a summary evaluation of pulmonary parenchyma, but an HRCT is still recommended.

It is also reported that there is an association between cancer and IIMs [83].

Association between polymyositis and dermatomyositis ranges from 6% to 60% and often are the early clinical manifestations of ovarian, renal, lung, and colorectal carcinomas and melanoma [84,85].

In the specific two big cohort national studies in Taiwan and Scotland, an elevated risk of lung, cervix uteri, and ovarian cancer was found in patients with DM, and for Hodgkin’s disease in patients with PM [84,86]. 

The risk of cancer in PM was the highest in younger patients and decreased with age.

In this case, the blood evaluation of myositis-specific autoantibodies (MSAs) can be useful.

In particular, the association between dermatomyositis with anti-TIF1γ and cancer is known [85]. Few studies report the correlation between other MSAs, such as anti-nuclear matrix protein (NXP2), anti-Jo-1, anti-Mi-2β, anti-HMGCR, and cancer in IIMs [87,88,89,90].

For all the abovementioned reasons, in this group of IIMs, MRI plays an important role because it can provide important information about the status of the disease, the response to therapies, and the pattern of muscle involvement, which are all important to guide toward the right diagnosis. In fact, more than in other immunological neuromuscular disorders, in IIMs, the symmetric or asymmetric and pattern of muscle involvement supports the diagnosis [49] (Table 1).

Therefore, whole-body MRI is becoming an important part of the diagnostic work-up, providing a complete examination of the muscle of the body (including suggestive pattern involvement) and improving the detection of cancer.

#### 4.4.1. Dermatomyositis

Dermatomyositis (DM) is a cellular immune-mediated disease that can affect derma, muscle, and systemic organs. It is correlated to a high risk in neoplasm development and it can also be caused by malignant tumors (paraneoplastic disease). 

It is possible to distinguish two forms of DM: one juvenile, more aggressive; and one of the adults. Females are more affected than males.

Clinically, it is possible to have dermic manifestations such as heliotropic rush, diffuse muscle pain, and weakness when the disease affects the muscle [91].

The pattern described on MRI is a symmetrical distribution of the muscles involved; the proximal ones are more affected than the distal ones, and frequent involvement of the anterior, posterior, and medial muscle groups of the thigh, as well as the frequent involvement of the quadriceps are described in [20,92].

Usually, in dermatomyositis, the inflammation infiltrates the subfascial and subcutaneous space and the interfascicular septa, which can explain the pattern of edema in the subcutaneous tissue, on the muscle periphery and honeycomb edema. 

The peripheral honeycomb edema could be explained by the underlying mechanism of complement-mediated vasculopathy of the small vessels characterizing DM [93].

When all these findings are present, it is strongly suggestive of dermatomyositis [26].

Myofascial involvement is a marker for rapidly progressive interstitial lung disease [94].

#### 4.4.2. Polymyositis

Polymyositis (PM) is a rare cellular immune-mediated inflammatory myopathy secondary to damage to the endomysium of the muscle [95]. Two peaks of incidence are described, between 5 and 15 years of age and 45 and 60 years of age [96].

The MRI pattern commonly describes symmetric muscle involvement, more often of the proximal muscle, in the thigh, where all the muscular groups can be affected, but a preferential involvement of the posterior flexor group is described in [20]. Usually, muscle edema has been described as “patchy” in DM and “diffuse” in PM [97]. 

Fascial edema is less frequent than dermatomyositis [98]. 

The presence of fascial edema on MRI is associated with the presence of myositis-specific antibodies (MSAs) and myositis-associated antibodies (MAAs) [26]. 

Subcutaneous fat edema is absent [26]. 

#### 4.4.3. Inclusion Body Myositis 

Inclusion body myositis (IBM) is the most common form of acquired myopathy in adults (>50 years) and is characterized by the presence of specific inclusions of amyloid-β protein in the muscle called inclusion bodies. Clinical onset is characterized by weakness and atrophy, mainly involving the quadriceps and deep finger flexor muscles in the forearm. It is described the involvement of more proximal muscles and dysphagia [25].

IBM shows an MRI pattern with an asymmetric fatty infiltration of skeletal muscles. Subcutaneous and fascial edema is usually not seen, and edema within the muscle is less prominent compared to the other IIMs [99]. 

The predominance of adipose infiltration compared to edema can be explained by the late onset of symptoms in patients with IBM when there is no longer a pattern of inflammation in the muscle, but instead adipose involution [20].

The muscles most frequently infiltrated with fat are the flexor digitorum profundus, anterior muscles of the thigh (with relative sparing of the rectus femoris), and all muscles of the leg, preferentially the medial part of the gastrocnemius [100].

The medial gastrocnemius muscle was described as the most affected muscle while a relative sparing of the tibialis posterior and soleus muscles [101].

In the thigh, a proximal-to-distal gradient of fat infiltration is observed for the adductors, quadriceps, sartorius, and medial gastrocnemius muscles [102]. 

The involvement of sartorius helps in identifying the disease since this muscle is usually sparing in the other idiopathic inflammatory myopathies [25].

#### 4.4.4. Immune-Mediated Necrotizing Myopathy

Immune-mediated necrotizing myopathy (IMNM) is a type of autoimmune myopathy characterized by the presence of two autoantibodies, those recognizing the signal recognition particle (SRP) and those targeting hydroxy-3-methylglutaryl-CoA reductase (HMGCR). These antibodies describe two different forms, but all cause the reclamation of immunological cells in the muscle, causing the necrosis of the muscle’s fiber [103].

It is a rare disease that usually affects children or young patients [104]. 

**Figure 6:**
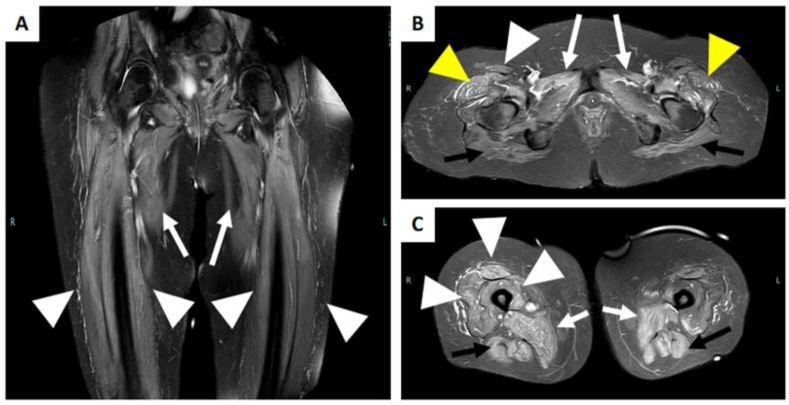
MRI of a patient with immune-mediated necrotizing myopathy with hydroxy-3-methylglutaryl-CoA reductase (HMGCR) antibodies positivity. (**A**) Coronal STIR image reveals a bilateral diffuse high intensity of adductors muscles (white arrow), and of the vasti muscle of the quadriceps femoris (with arrowhead). (**B**) Axial STIR image shows a bilateral and diffuse high intensity of the gluteus maximum muscle (black arrow), of the adductor muscles (white arrow), of the tensor of fascia lata (yellow arrowhead), and the right rectus femoris muscle (white arrowhead). (**C**) Axial STIR image shows bilateral involvement of the adductor muscles (white arrow), the hamstring muscles (black arrow), and the vasti muscle and rectus femoris of the right quadriceps femoris muscle (white arrowhead).

MRI imaging is able to describe the edema with a prevalent involvement of the lumbar paraspinal muscles, gluteus, medius, gluteus minimus, adductor magnus, and hamstrings muscles (Figure 6).

MRI can offer a sensitive biomarker to predict prognosis and monitor disease activity after therapy administration [105].

#### 4.4.5. Focal Myositis

Focal myositis (FM) is a particular kind of benign inflammatory disorder confined to an entire single muscle or a part of it, or other multiple adjacent muscles [106]. The etiopathogenesis of this disorder is not completely understood, but there is a possible role of a combination of triggering factors such as dysimmune diseases or denervation in patients with genetic predisposition [107].

In these disorders, MRI is extremely useful because it is necessary to isolate the single muscles, and because of these disorders, it can sometimes appear as a neoplasm and is necessary for differential diagnosis [108].

FM appears on MRI as a circumscribed mass within the muscle or an enlargement of the muscle involved. Adjacent structures are spared by the mass but can show a high intensity on fluid-sensitive images due to the associated edema [109].

At times, the muscle is technically and histologically difficult to evaluate (e.g., orbital myositis), and so, imaging and MRI are the only tools available in such cases [110].

## 5. Conclusions

Whole-body MRI allows for the diagnosis of active and chronic muscle disease through a full assessment of the whole body’s musculature, allowing for the identification of disease patterns and is thus a great tool for both clinical practice and research. In fact, the most current knowledge of muscle involvement in neuromuscular diseases is limited for the study of the lower limbs. This necessitates a thorough and complete study of any patterns that may specifically be associated with an acquired neuromuscular disorder, as well as any that has already occurred for inherited neuromuscular diseases.

Whole-body MRI is helpful for the identification of the site of active disease to orient a biopsy for definitive histologic diagnosis, and it represents a promising tool for the evaluation of the response of these diseases to treatment. Scanning the whole body is a good imaging technique to identify systemic involvement or cancer usually associated with NMDs.

## Figures and Tables

**Table 1:** Different features on MRI of the most common IIMs.

	POLYMYOSITIS	DERMATOMYOSITIS	IBM
SIDE INVOLVEMENT	Symmetric	Symmetric	Asymmetric
MUSCLE GRADIENT	Proximal predominance	Proximal predominance	Distal predominance
PATTERN OF EDEMA	Diffuse homogeneous	Peripheral distribution, patchy or honeycomb pattern	Less frequent than fat degeneration
PATTERN OF MUSCLE FAT INFILTRATION	Less frequent than edema	Less frequent than edema	Predominant fatty infiltration
FASCIAL INVOLVEMENT	Not always, less than dermatomyositis	Present	Absent
SUBCUTANEOUS INVOLVEMENT	Absent	Significant subcutaneous edema	Absent
MUSCLE MORE INVOLVED	Thigh with global or posterior compartment involvement	Thigh with anterior compartment involvement (quadriceps)	medial head of gastrocnemius, flexor digitorum profundus, anterior compartment of the thigh

## Data Availability

Not applicable.
